# Female patients with vascular disease receive less medical optimization despite more health care utilization

**DOI:** 10.1016/j.jvs.2025.09.054

**Published:** 2025-10-09

**Authors:** Mikayla N. Lowenkamp, Hasan Nassereldine, Alexas Iams, Edith Tzeng, Katherine M. Reitz

**Affiliations:** a Division of Vascular Surgery, University of Pittsburgh, Pittsburgh;; b Department of Surgery, University of Pittsburgh, Pittsburgh;; c Department of Surgery, Veterans Affairs Pittsburgh Healthcare System.

**Keywords:** Sex, Disparities, Medical, Optimization

## Abstract

**Objective:**

Despite optimal medical therapy (OMT) being a cornerstone of vascular care, female patients are less likely to receive it in comparison with males. This study aimed to quantify preoperative access to care and hypothesized that women would have lower documented OMT prescription rates while controlling for health care use.

**Methods:**

This retrospective cohort study used a single-center, multihospital dataset of all adults undergoing index, elective, operative interventions for carotid, aortic, or lower extremity disease (2016–2024). The primary outcome was documented OMT (statin, antiplatelet, and nonsmoking). Multivariable logistic regression evaluated the association between documented OMT and sex, adjusting for comorbidities and health care use.

**Results:**

Among 18,673 patients, 8135 (43.6%) were female. Females more frequently saw a primary care physician (PCP) (42.0% vs 40.5%; *P* = .042) with more visits on average (3.3 ± 7.4 vs 2.9 ± 7.0; *P* < .001). Females were less likely to see a cardiologist (28.9% vs 38.7%; *P* < .001). Despite increased PCP exposure, females were less frequently prescribed OMT (23.0% vs 28.2%; *P* < .001). They were more frequently prescribed pain medications (50.5% vs 42.9%; *P* < .001). On multivariable modeling, female sex had lower odds of preoperative OMT (adjusted odds ratio [aOR], 0.80; 95% confidence interval [CI], 0.75–0.6; *P* < .001). A PCP visit (aOR, 1.63; 95% CI, 1.52–1.75; *P* < .001) or cardiology visit (aOR, 1.73; 95% CI, 1.60–1.87; *P* < .001) were both associated with an increased rate of achieving documented OMT.

**Conclusions:**

Female patients were less likely to have documented OMT despite increased health care access in the form of PCP visits. Our findings highlight a potential provider bias leading to an undertreatment of females with vascular disease.

Peripheral artery disease (PAD) was previously thought of as a pathology with male predominance.^1,2^ However, the global disease burden estimates demonstrate an equal if not greater prevalence of PAD in female patients—a fact frequently unrecognized by both clinicians and patients.^3^ This historical bias has resulted in the chronic under-representation of female patients in comparative effectiveness research and clinical trials, which then exacerbates the underdiagnosis and undertreatment of PAD in females.^4–7^ One example of the undertreatment of PAD in female patients is the lower rates of guideline-directed medical therapies or optimal medical therapy (OMT) in the form of antiplatelet agents, high-intensity statins, and smoking cessation.^7,8^

The etiology for the disparate rates of OMT between the sexes is unknown. However, several hypotheses exist, one being a bias among providers who underestimate the cardiovascular risk or underdiagnose PAD in the female patients.^8,9^ Second, evidence suggests hesitancy may exist in the receipt of numerous medical therapies and a deference toward avoiding potential side effects or preferences for natural remedies in female patients.^10^ Third, there are hypothesized differences in health care use.^10–12^ Female patients have differing access to care and preferences for interventions in comparison with their male counterparts, resulting in unequal opportunities to receive OMT.

This study aimed to explore the drivers of disparities, including potential provider biases, that may contribute to disparate rates of documented OMT between sexes. We hypothesized that female patients would demonstrate lower rates of documented OMT irrespective of health care use.

## METHODS

This is a retrospective cohort study conducted in a large, single health care system serving 18 academic and community hospitals, identifying patients undergoing abdominal aortic aneurysm (AAA) repair and carotid as well as lower extremity artery revascularization procedures. This study used deidentified data and thus was deemed exempt from informed consent by the University of Pittsburgh Institutional Review Board (STUDY24090124). The STROBE reporting guidelines were followed.^13^

### Data source and patient cohort.

All patients who underwent surgical procedures for AAA, carotid artery, or lower extremity vascular disease were identified in the electronic health record (EHR) via validated surgical Current Procedural Terminology codes.^14,15^ Patients were included if they underwent an open or endovascular AAA repair, a carotid stenting or carotid endarterectomy, or lower extremity endovascular and/or open surgical revascularization procedures with corresponding elective *International Classification of Diseases*, Clinical Modification, 10th Revision (ICD-10) codes. We included only the index operation for each patient. Patients were excluded if they were under the age of 18, had their procedure at the Children’s Hospital of Pittsburgh, or presented emergently (as a trauma activation or with urgent/emergent operative status).

Using structural query language, preoperative, periprocedural, and postoperative data from both inpatient Cerner Millennium (Cerner Corporation) and outpatient Epic (Epic Systems Corporation) EHR systems were used at our institution. Comorbidities such as hypertension, diabetes, coronary artery disease (CAD), chronic obstructive pulmonary disease, and end-stage renal disease were identified using ICD-10 codes recorded in the outpatient and inpatient EHRs. Race was abstracted from the structured, self-reported outpatient EHR fields. Neighborhood-level disadvantage was quantified using the Area Deprivation Index (ADI) based on residential zip codes documented at the index hospital admission.^16^ Additional surgical characteristics were abstracted from structured anesthesia records (ie, anesthesia type, urgency).

Patients were dichotomized by sex assigned at birth as per documentation in the EHR. Our EHR only recently began documenting gender; therefore, for this report, the exposure is limited to sex and not gender identity. Health care use was defined as outpatient primary care physician (PCP) visits or cardiology visits in the year before the index procedure.

### Outcomes.

The primary outcome of interest was documented, preoperative OMT. The composite outcome of OMT was defined by the inclusion of all three items: an antiplatelet agent, a statin, and nonsmoking at the time of the operation as per the American College of Cardiology (ACC) and American Heart Association (AHA) guidelines.^17^ Antithrombotic (ie, aspirin, P2Y12 inhibitor) and statin therapies (ie, low, medium, and high intensities) were quantified based on their presence in outpatient medical reconciliations or EHR-generated prescriptions within the 90 days before the index intervention. Nonsmoking status (ie, former/nonsmoker) was quantified based on patient report documented within the outpatient EHR. Secondary outcomes included each individual item comprising OMT and high-intensity statins specifically. Chart abstraction of documented pain medications (ie, prescription nonsteroidal anti-inflammatory drugs, antidepressants, opioids, topical pain medications, and gabapentinoids) was conducted in the same manner as for OMT therapies. We included all prescription pain medication modalities used for the treatment of chronic pain in accordance with the anesthesia practice guidelines.^18,19^

### Statistical analyses.

Continuous variables were presented as mean ± standard deviation or median (interquartile range). Categorical variables were presented as frequencies (percent). Comparisons between continuous variables were made using either the Student *t* test or the Mann-Whitney *U* test, where appropriate. Comparisons between categorical variables were completed using either *χ*^2^ or Fisher’s exact, where appropriate. We quantified missing data, which is presented in Supplementary Table I (online only).

Multivariable logistic regression models were used to evaluate the likelihood of our primary outcome (composite OMT and its individual items) based on a priori clinically relevant variables with a robust variance estimator to account for the large dataset and possible misclassification. Multivariable models adjusted for age, race, comorbidities such as diabetes, CAD, and chronic obstructive pulmonary disease, ADI, operative type, and preoperative PCP and cardiology visits. This generated adjusted odds ratios (aOR) with 95% confidence intervals (95% CIs). The heterogeneity of treatment effect was evaluated using interaction terms to determine differences among a priori clinically relevant subgroups.^20^ Subgroups included age (<65 years vs ≥65 years), race (White vs non-White race), preoperative PCP and cardiology visits, and operation type.

Analyses were performed using StataMP version 19.0 (StataCorp). All tests were two sided, and a *P* value of less than .05 was considered statistically significant.

### Sensitivity analysis.

To assess the robustness of our findings, we conducted three sensitivity analyses. First, we excluded patients who underwent an abdominal aortic procedure, because the indication for statin in this case, per ACC/AHA guidelines, is largely dependent on provider’s review of preoperative imaging and physical exam findings which cannot be quantified with Current Procedural Terminology codes.^21^ In our second sensitivity analysis, we defined OMT as only antiplatelet therapy and statin prescriptions, which are dependent only on provider action and not patient compliance. Third, we identified those who presented with ICD-10 diagnosis codes for fibromuscular dysplasia with subsequent carotid revascularization or popliteal aneurysms and subsequent lower extremity revascularizations. We then excluded them from our analysis because the indication for statin prescribing may be unclear.

## RESULTS

There were 18,673 patients who met all the inclusion and exclusion criteria with an average age of 66.3 ± 13.0 years; 8135 (43.6%) female and 16,765 were White (90.0%) White. The most common procedure was a carotid surgery (n = 9631 [51.6%]), followed by lower extremity revascularization (n = 7125 [38.2%]) and AAA repair (n = 1917 [10.3%]).

### Baseline characteristics.

Baseline characteristic data are summarized in Table I. At baseline, females were younger (64.7 ± 14.2 years vs 67.5 ± 11.8 years; *P* < .001) and less likely to self-identify as White (88.3% vs 90.9%; *P* < .001). Females had fewer baseline comorbidities, specifically lower rates of hypertension, diabetes, CAD, chronic obstructive pulmonary disease, and end-stage renal disease in comparison with males. Female patients were more likely to be on prescription pain medication at the time of their operation (50.5% vs 42.9%; *P* < .001). Although female patients had a statistically significantly greater ADI, in terms of clinical impact, the difference was relatively small (71.3 ± 15.8 vs 70.4 ± 16.1; *P* < .001). The disease patterns differed between the sexes; females less commonly underwent an operation for an AAA (5.0% vs 14.3%) or lower extremity disease (33.1% vs 42.1%), but more commonly underwent a procedure for carotid artery disease (61.9% vs 43.6%) (Table I) (*P* < .001).

We observed differences among OMT overall and among its individual items across the sexes (Table II). Females less commonly achieved preoperative OMT (23.0% vs 28.2%; *P* < .001). Females received fewer antiplatelet agents (42.7% vs 47.5%; *P* < .001) and fewer statins (47.4% vs 55.7%; *P* < .001). Moreover, although the rates of high-intensity statins were low between both cohorts, females less frequently received them than their male counterparts (23.5% vs 30.8%; *P* < .001). Females were more commonly active smokers at the time of their operation (20.8% vs 19.2%; *P* = .007).

### Health care use.

**Fig 1.**
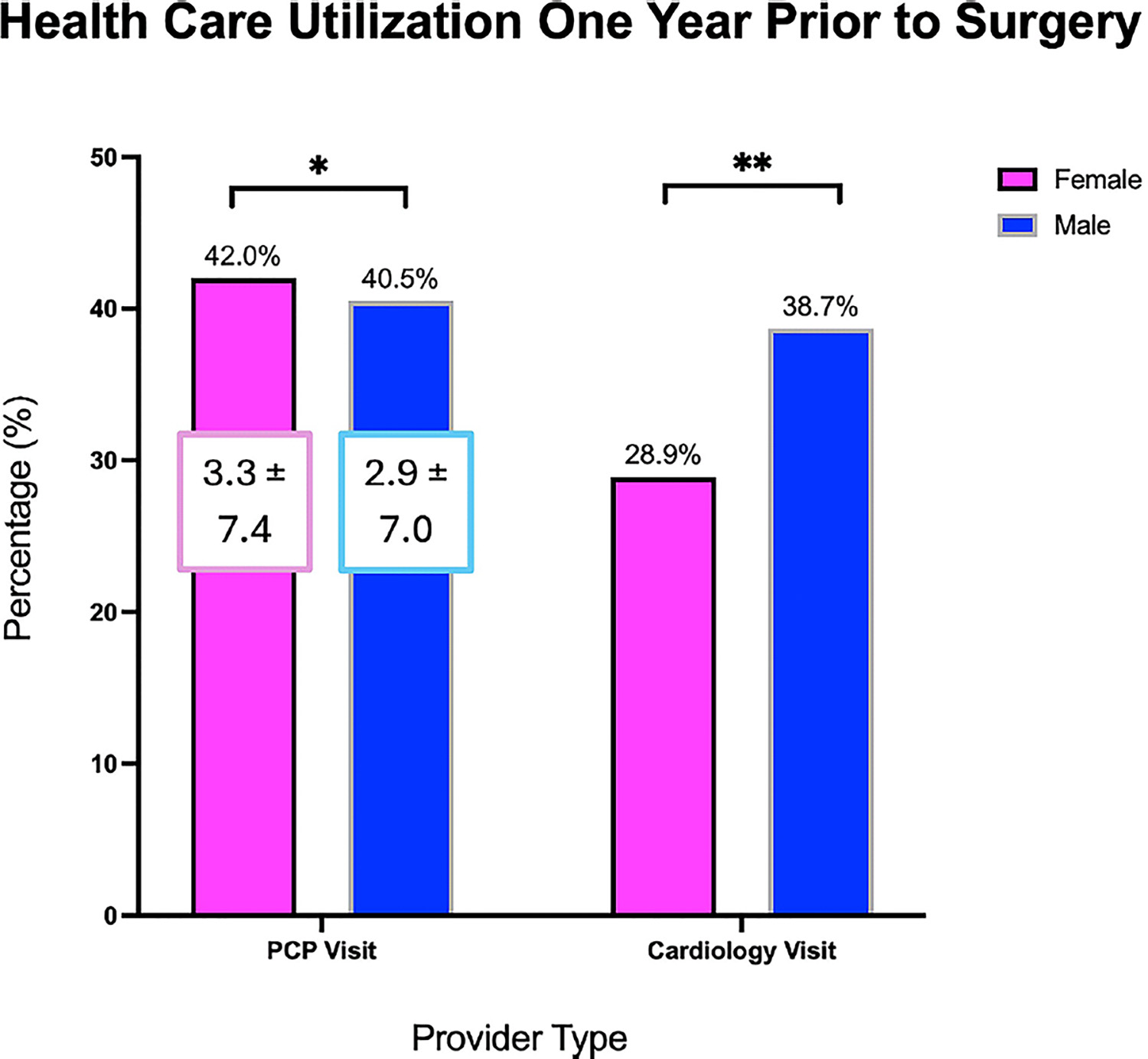
Health care use in the year before surgery by sex. *PCP*, primary care physician.

Preoperative health care use varied by sex (Fig 1). Females were more commonly seen by their PCP in the year before surgery in comparison with males (42.0% vs 40.5%; *P* = .042) and saw their PCP more frequently in that year (3.3 ± 7.4 vs 2.9 ± 7.0 visits; *P* < .001). Females were less commonly seen by a cardiologist preoperatively (28.9% vs 38.7%; *P* < .001).

### Predictors of documented OMT prescription.

**Fig 2.**
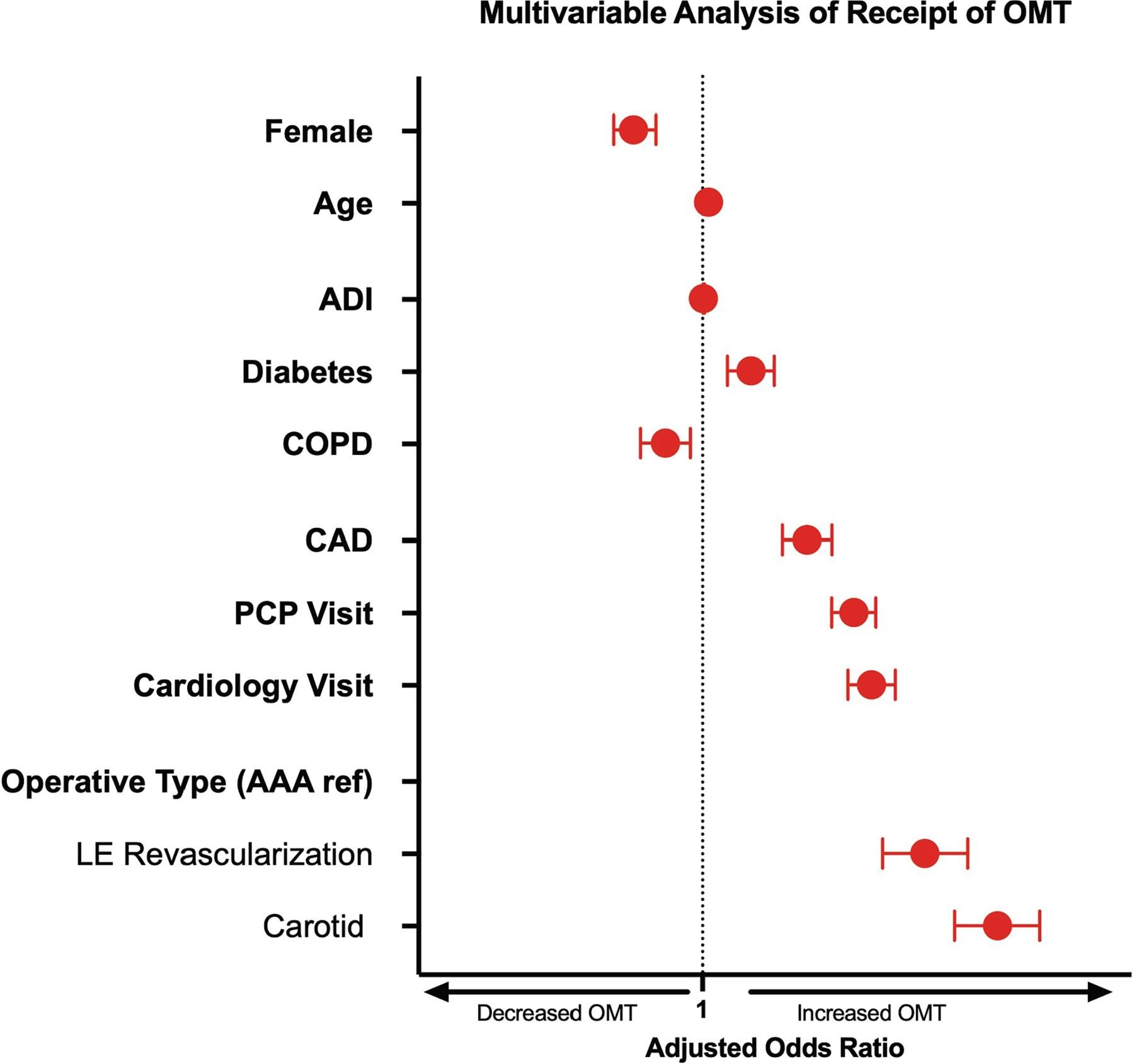
Multivariable logistic regression modeling receipt of optimal medical therapy (*OMT*) controlling for race with robust variance estimation. *Red* indicates statistically significant variable. *AAA*, abdominal aortic aneurysm; *ADI*, Area Deprivation Index; *CAD*, coronary artery disease; *COPD*, chronic obstructive pulmonary disease; *LE*, lower extremity; *PCP*, primary care physician.

On multivariable logistic regression, females were 20% less likely to achieve documented OMT (aOR, 0.80; 95% CI, 0.75–0.86; *P* < .001) while controlling for age, race, comorbidities, ADI, health care use, and operative procedure (Fig 2). In the same model, both preoperative PCP (aOR, 1.63; 95% CI, 1.52–1.75; *P* < .001) and cardiologist (aOR, 1.73; 95% CI, 1.60–1.87; *P* < .001) encounters increased the odds of achieving documented OMT.

For each secondary outcome, female sex was again associated with a reduced adjusted risk of documented antiplatelet therapy (aOR, 0.84; 95% CI, 0.79–90; *P* < .001) or statin therapy (aOR, 0.78, 95% CI, 0.73–0.83; *P* < .001) (Supplementary Table II, online only) when compared with males. Sex was not associated with differences in smoking cessation (aOR, 1.01, 95% CI, 0.93–1.09; *P* = .86) (Supplementary Table II, online only)

### Subgroup analyses.

**Fig 3.**
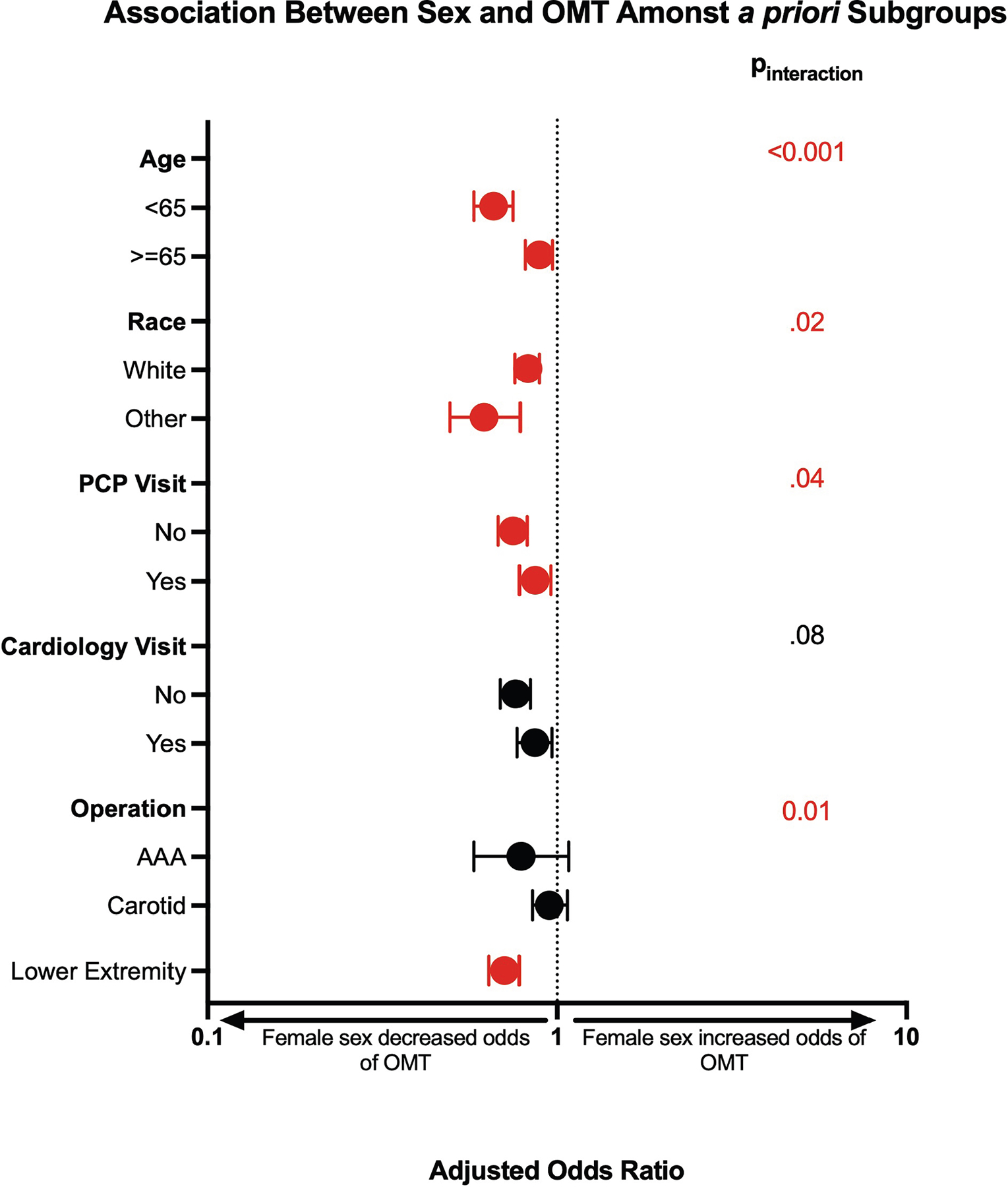
Subgroup analysis on receipt of optimal medical therapy (*OMT*) among subgroups. *AAA*, abdominal aortic aneurysm; *ADI*, Area Deprivation Index; *PCP*, primary care physician.

The association between female sex and a lower risk of achieving OMT was ubiquitously observed amongst the subgroups (Fig 3). However, heterogeneity of treatment effect was observed. The lower adjusted risk of documented OMT for females was more pronounced among younger females when compared with older females (*P*_interaction_ < .001) and non-White race compared with White race (*P*_interaction_ = .02) and those who did not see a PCP (*P*_interaction_ = .04). The association between female sex and a lower risk of documented OMT was most significant among those undergoing lower extremity revascularization when compared with other procedures (*P*_interaction_ = .01).

### Sensitivity analyses.

All sensitivity analyses yielded the same result: female patients had lower adjusted odds of receiving OMT while controlling for comorbidities, health care use, and procedure. These models can be found in Supplementary Tables III and IV (online only). Upon exclusion of aortic repairs from the cohort, we find that females still had decreased odds of receiving OMT (aOR, 0.80; 95% CI, 0.74–0.87; *P* < .001) (Supplementary Tables III, online only). Limiting the definition of OMT to only antiplatelet and statin agents, which require provider initiation, to be considered medically optimized, we again find that females had lower adjusted odds of documented OMT (aOR, 0.79; 95% CI, 0.74–0.84; *P* < .001) (Supplementary Table IV, online only). Upon excluding patients with fibromuscular dysplasia or popliteal aneurysms, female sex continued to be associated with a 20% lower rate of documented OMT (aOR, 0.80; 95% CI, 0.74–0.86; *P* < .001), whereas preoperative PCP (aOR, 1.64; 95% CI, 1.52–1.76; *P* < .001) and cardiology (aOR, 1.74; 95% CI, 1.61–1.88; *P* < .001) encounters continued to be associated with increased documented OMT.

## DISCUSSION

Female patients are frequently reported to be undertreated for atherosclerotic cardiovascular diseases.^6,7^ In this study, we found that female patients had a lower adjusted odds of documented OMT at the time of their elective operation. The association between female sex and lower adjusted risk of documented OMT continued throughout subgroups and was present for each item of OMT, except smoking cessation. Interestingly, females had more frequent health care use, which was associated with an increased likelihood of documented OMT. However, even with more frequent primary care visits, females remained less likely to receive OMT when compared with males. These findings suggest the disparate documented OMT rates between sexes are not due to health care access, but may reflect a potential provider bias in diagnosing and managing atherosclerotic cardiovascular disease in female patients.

The ACC/AHA guidelines recommend that all patients with PAD be maintained on an antiplatelet agent, a high-intensity statin, and achieve smoking cessation. However, prior work has documented that males are 50% more likely to be prescribed antiplatelet agents in comparison with females for atherosclerotic disease.^22^ Even after vascular intervention, females remain less likely to be prescribed these guideline-directed medical therapy (GDMT). Moreover, when they are prescribed GDMT, females are less likely to meet treatment targets such as lipid levels, blood glucose, and blood pressure goals.^23^ Similar disparities are seen in females with CAD.^24^ In our cohort, patients with concomitant CAD had an increased likelihood of documented OMT, suggesting that the importance of medical therapy for PAD may be underemphasized. Although our data cannot directly assess the underdiagnosis of cardiovascular disease, we agree with prior work suggesting that females may be underdiagnosed with atherosclerotic cardiovascular disease, which could partly explain lower OMT documentation.^25^ Importantly, sex disparities in the management of atherosclerotic disease spans multiple countries and specialists, with consistently worse outcomes for female patients.^22,26^

A current hypothesis for the lower documented OMT rates in female patients is provider bias. Physician awareness of PAD is low and historical sex bias has led to decreased screening and risk factor management in females, who were previously thought to be less affected by atherosceltosis.^27,28^ Survey data have shown that, when provided are provided with two identical patients differing only by sex, physicians consistently underestimate cardiovascular risks in the female patient.^29^ The combination of low awareness of PAD prevalence in females and systematic underestimation of their risk contributes to chronic undertreatment leading to disease progression and profound morbidity and mortality.^6,7,28^ Other possible contributors included sex-based differences in medication adherence and provider preferences for alternative drug classes in female patients.^30,31^

Antithrombotic and lipid-lowering therapy prescribing is simple; therapies have broad dosing ranges without toxicity, and the side effects are easily managed with a low rate of adverse events.^32–35^ Smoking cessation counseling carries essentially no risk, and therapies to increase the rate of smoking cessation are safe and straightforward. Therefore, a broad set of providers should feel comfortable prescribing these inexpensive and safe therapies. In our study, PCP and cardiology evaluations in the year before surgical interventions increased the rate of documented OMT. However, subgroup analyses confirmed that even females actively engaged in the health care system remained undertreated. Patient-based preferences may affect female’s willingness to receive prescribed medications, with certain studies documenting a preference for natural remedies.^10,12^ However, our data show that females had a higher rate of prescription pain medication, which may indicate a willingness to engage with pharmacological treatment. Although we recognize that all medications are not perceived equally, the increased pain medication prescription among female patients suggests the lower documented OMT rates are unlikely to be explained solely by patient reluctance.

We only included elective interventions in our cohort; thus, all patients were evaluated by a vascular specialist before surgery. This underscores a critical missed opportunity: although systematic and specialty-wide disparities exist, the vascular community is not exempt. Vascular specialists are uniquely positioned to identify patients lacking GDMT and initiate or coordinate its implementation. The low OMT rates observed in our cohort highlight the urgent need for vascular providers to adopt a more proactive role in medical optimization. This disparity is most prominent in those pursuing lower extremity revascularization, where the data for OMT are the strongest to decrease adverse limb outcomes and mortality.^14,36^ Vascular specialists must take this opportunity, especially before a surgical intervention, to review patient medications and ensure OMT is considered and prescribed.

Implementation science offers strategies to improve the delivery of evidence-based therapies.^37,38^ Our data suggest that the passive implementation strategy—-relying solely on guideline enactment—is insufficient, especially among females. Active implementation strategies, developed in collaboration with implementation experts are needed nationally and locally to improve OMT delivery.^38^

The limitations of this study are inherent primarily to its retrospective nature. The large dataset limited the granularity of the patient encounters, and we relied heavily on accurate EHR documentation of procedures, diagnoses, smoking status, and medications. Despite low documented missingness, there remains an ongoing risk of misclassification, particularly with medications like aspirin, which can be obtained over the counter. Although the EHR platform is consistent across hospitals, the availability of smoking cessation programs varies and thus we defined OMT by preoperative smoking status. Our methods cannot capture or inform patient medication compliance, which may also differ between the sexes, or intolerances and allergies. Further, sex and gender are two distinct entities, and the impact of gender on OMT could not be explored. Last, we were unable to explore other potential bias, inducing sources including patient medication adherence, provider preferences, and the impact of the sex of the vascular specialists, which may also vary across sexes.^30,31^

In this large cohort of patients undergoing elective PAD interventions, females had similar health care use as males but significantly lower documented OMT. These findings highlight a potential provider bias in the treatment of PAD. Addressing these disparities requires greater clinical awareness of the prevalence, presenting symptoms, and management of PAD in female patients. In addition, more sex-specific research would allow providers to tailor medical therapy to optimize outcomes for female patients, which begins with improved inclusion and recruitment of females in PAD studies. For now, the vascular surgeon-patient encounter provides a meaningful opportunity to medically optimize our patients, and we should aggressively pursue risk factor identification and modification.

## CONCLUSIONS

Recent data suggest that the prevalence of vascular disease is equal between the sexes; however, this disparity is still unrecognized by health care providers and patients. As such, female patients are less likely to receive GDMT despite increased health care access in the form of PCP visits. This demonstrates a potential provider bias that leads to the undertreatment and mismanagement of female patients with vascular disease. Although these disparities are widely reported across specialties, our study emphasizes that vascular providers remain a critical point of failure in medical optimization. These findings point to an urgent need for greater accountability and systematic change within the vascular community for proactive initiation and coordination of medical management, particularly for female patients. Future work should focus on implementation strategies to improve consistency and equity of preventative cardiovascular care.

## Supplementary Material

sup1

sup2

sup3

sup4

## Figures and Tables

**Table I.** Baseline characteristics, comorbidities, medication use, and disease distribution by sex

	Female (n = 8140)	Male (n = 10,579)	*P* value
Age, years	64.7 ± 14.2	67.5 ± 11.8	<.001
Race			<.001
White	7187 (88.3)	9578 (90.5)	
Black	732 (9.0)	663 (6.3)	
Other	69 (0.9)	90 (0.9)	
Not specified	147 (1.8)	207 (2.0)	
Comorbidities			
Hypertension	4786 (59.2)	6824 (65.1)	<.001
Diabetes	2359 (29.1)	3771 (35.8)	.043
CAD	2560 (31.6)	5220 (49.7)	<.001
Chronic obstructive pulmonary disease	2034 (25.1)	2883 (27.4)	<.001
End-stage renal disease	765 (9.4)	1395 (13.3)	<.001
Disease type			
AAA	404 (5.0)	1513 (14.3)	<.001
Carotid artery disease	5038 (61.9)	4593 (43.6)	
Lower extremity disease	2693 (33.1)	4432 (42.1)	
Insurance status			<.001
Commercial	1931 (23.7)	2102 (19.9)	
Medicaid	1041 (12.8)	902 (8.5)	
Medicare	5092 (62.6)	7143 (67.7)	
Self-pay/other	76 (0.9)	409 (3.9)	

*AAA*, Abdominal aortic aneurysm; *CAD*, coronary artery disease.

Values are mean ± standard deviation or number (%).

**Table II.** Optimal medical therapy (OMT) composite and individual component rates between sexes

	Female (n = 8140)	Male (n = 10,579)	*P* value
Current smoker	1673 (21)	2011 (19)	.007
Preoperative medications			
Antiplatelet agents	3470 (43)	5004 (48)	<.001
Anticoagulation	1016 (13)	1710 (16)	<.001
Statin	3857 (47)	5912 (56)	<.001
High-intensity statin	1911 (24)	3246 (31)	<.001
OMT	1867 (23.0)	2972 (28.2)	<.001
Pain medications	4105 (51)	4519 (43)	<.001
Prescription NSAID/acetaminophen	1873 (23.0)	2052 (19.5)	<.001
Gabapentinoids	1494 (18.4)	1542 (14.6)	<.001
Tricyclic antidepressants/SNRI	911 (11.2)	642 (6.1)	<.001
Narcotics	1920 (23.6)	2343 (22.2)	.027
Muscle relaxants	780 (9.6)	675 (6.4)	<.001
Topical agents	479 (5.9)	398 (3.8)	<.001

*NSAID*, Nonsteroidal anti-inflammatory; *SNRI*, serotonin-norepinephrine reuptake inhibitors.

Values are number (%).

## ARTICLE HIGHLIGHTS

- **Type of Research:** Single-center retrospective cohort study
- **Key Findings:** Among 18,673 patients undergoing elective vascular surgery operations, female patients were less likely to be prescribed optimal medical therapy for peripheral artery disease despite having more interactions with the health care system.
- **Take Home Message:** Provider biases may exist in the treatment of female patients with vascular disease.
