# Supplementary material for: Female patients with vascular disease receive less medical optimization despite more health care utilization
Source: J Vasc Surg. Author manuscript; Available in PMC 2026 Apr 5. (PMC13050515; doi:10.1016/j.jvs.2025.09.054)
Supplement: sup1 [file NIHMS2161498-supplement-sup1.pdf]

Supplementary Table IV (online only) Multivariable logistic regression where optimal medical therapy (OMT) only contained antiplatelet agent and statin (smoking cessation was removed)

Variable	aOR	[95% CI]		*P* value
Female sex	0.79	0.74	0.84	<.001
Age	1.00	1.00	1.01	<.001
Race				
White	1.92	0.79	4.75	.15
Black	1.86	0.75	4.61	.18
Asian	1.90	0.71	5.08	.20
Not specified	2.42	0.95	6.15	.06
ADI	1.01	1.01	1.01	<.001
DM	1.06	0.99	1.14	.10
COPD	1.14	1.06	1.223	<.001
CAD	1.37	1.27	1.47	<.001
PCP visit	1.72	1.61	1.84	<.001
Cardiology visit	1.81	1.68	1.95	<.001
Operation (ref AAA)				

Variable	aOR	[95% CI]		*P* value
Lower extremity	2.46	2.16	2.79	<.001
Carotid	2.60	2.29	2.95	<.001

ADI, **Area Deprivation Index;** ***aOR,*** **adjusted odds ratio;** ***CAD,*** **coronary artery disease;** ***CI,*** **confidence interval;** ***COPD,*** **chronic obstructive pulmonary disease;** ***DM,*** **diabetes mellitus;** ***PCP,*** **primary care physician.**
