# Supplementary material for: Female patients with vascular disease receive less medical optimization despite more health care utilization
Source: J Vasc Surg. Author manuscript; Available in PMC 2026 Apr 5. (PMC13050515; doi:10.1016/j.jvs.2025.09.054)
Supplement: sup2 [file NIHMS2161498-supplement-sup2.pdf]

Supplementary Table I (online only) Missingness table summarizing missingness of key variables

Variable	Percent missing
Age	0
Race	0
Comorbidities	
Hypertension	0.3
Diabetes	0.3
CAS	0.3
Chronic obstructive pulmonary disease	0.3
End-stage renal disease	0.3
Operative type	0
Smoking status	0.9
Antiplatelet	0
Statin	0
ADI	4.3
PCP visit	0
Cardiology visit	0

***ADI*, Area Deprivation Index; *CAD*, coronary artery disease.**
