# Supplementary material for: Female patients with vascular disease receive less medical optimization despite more health care utilization
Source: J Vasc Surg. Author manuscript; Available in PMC 2026 Apr 5. (PMC13050515; doi:10.1016/j.jvs.2025.09.054)
Supplement: sup3 [file NIHMS2161498-supplement-sup3.pdf]

# Supplementary Table III (online only) Logistic regression excluding abdominal aortic aneurysm (AAA) repairs, including carotid and lower extremity revascularizations

| Variable | aOR | 95% CI | | *P* value |
|---|---|---|---|---|
| Female sex | 0.80 | 0.744 | 0.87 | <.001 |
| Age | 1.02 | 1.02 | 1.02 | <.001 |
| Race | | | | |
| White | 1.30 | 0.51 | 3.28 | .58 |
| Black | 1.13 | 0.44 | 2.89 | .79 |
| Asian | 1.42 | 0.51 | 3.95 | .50 |
| Not specified | 1.50 | 0.57 | 3.93 | .42 |
| ADI | 1.00 | 1.00 | 1.00 | .003 |
| DM | 1.19 | 1.10 | 1.29 | <.001 |
| COPD | 0.88 | 0.80 | 0.95 | .002 |
| CAD | 1.34 | 1.23 | 1.50 | <.001 |
| PCP visit | 1.66 | 1.54 | 1.79 | <.001 |
| Cardiology visit | 1.75 | 1.62 | 1.90 | <.001 |
| Operation (ref lower extremity) | | | | |
| Carotid | 1.26 | 1.17 | 1.36 | <.001 |

***AAA*, Abdominal aortic aneurysm; *ADI*, Area Deprivation Index; *aOR*, adjusted odds ratio; *CAD*, coronary artery disease; *CI*, confidence interval; *COPD*, chronic obstructive pulmonary disease; *DM*, diabetes mellitus; *PCP*, primary care physician.**
