# Supplementary material for: Female patients with vascular disease receive less medical optimization despite more health care utilization
Source: J Vasc Surg. Author manuscript; Available in PMC 2026 Apr 5. (PMC13050515; doi:10.1016/j.jvs.2025.09.054)
Supplement: sup4 [file NIHMS2161498-supplement-sup4.pdf]

Supplementary Table II (online only) Multivariable logistic regression models of each individual components of optimal medical therapy (OMT) (antiplatelet agent, statin, smoking cessation)

Variable	aOR	95% CI		*P* value
Antiplatelet agent				
Female sex	0.84	0.79	0.90	<.001
Age	1.01	1.00	1.01	<.001
Race				
White	2.45	1.11	5.39	.03
Black	2.38	1.07	5.27	.03
Asian/Pacific Islander	1.81	0.76	4.36	.18
Not specified	3.01	1.32	6.84	.01
ADI	1.01	1.01	1.01	<.001
Diabetes	1.01	0.95	1.08	.714
COPD	1.13	1.05	1.21	.001
CAD	1.35	1.25	1.44	<.001
PCP visit	1.23	1.15	1.31	<.001
Cardiology visit	1.67	1.56	1.79	<.001

Variable	aOR	95% CI		*P* value
Operative type (AAA ref)				
Lower extremity	2.94	2.62	3.30	<.001
Carotid	2.92	2.60	3.28	<.001
Statin				
Female sex	0.78	0.73	0.83	<.001
Age	1.02	1.02	1.02	<.001
Race				
White	1.06	0.47	2.40	.895
Black	0.94	0.41	2.14	.876
Asian/Pacific Islander	0.99	0.40	2.42	.974
Not specified	1.18	0.50	2.76	.708
ADI	1.00	1.00	1.01	<.001
Diabetes	1.25	1.17	1.35	<.001
COPD	1.16	1.07	1.25	<.001
CAD	1.18	1.10	1.27	<.001
PCP visit	3.30	3.09	3.53	<.001
Cardiology visit	2.12	1.97	2.28	<.001

Variable	aOR	95% CI		*P* value
Operative type (AAA ref)				
Lower extremity	1.34	1.20	1.50	<.001
Carotid	1.34	1.20	1.50	<.001
Smoking cessation				
Female sex	1.01	0.93	1.09	.853
Age	1.04	1.04	1.04	<.001
Race				
White	0.60	0.22	1.64	.322
Black	0.52	0.19	1.42	.201
Asian/Pacific Islander	1.37	0.42	4.47	.605
Not specified	0.60	0.21	1.68	.327
ADI	0.99	0.99	0.99	<.001
Diabetes	1.44	1.32	1.58	<.001
COPD	0.43	0.39	0.46	<.001
CAD	1.23	1.12	1.35	<.001
PCP visit	1.07	0.99	1.17	.082
Cardiology visit	1.04	0.95	1.14	.374

Variable	aOR	95% CI		*P* value
Operative type (AAA ref)				
Lower extremity	0.62	0.54	0.72	<.001
Carotid	1.09	0.94	1.26	.245

AAA, **Abdominal aortic aneurysm;** ***ADI,*** **Area Deprivation Index;** ***aOR,*** **adjusted odds ratio;** ***CAD,*** **coronary artery disease;** ***CI,*** **confidence interval;** ***COPD,*** **chronic obstructive pulmonary disease;** ***PCP,*** **primary care physician.**

All variables were modeled with a robust variance estimator.
